# Interleukin-17A derived from mast cells contributes to fibrosis in gastric cancer with peritoneal dissemination

**DOI:** 10.1007/s10120-020-01092-2

**Published:** 2020-06-02

**Authors:** Katsuya Gunjigake, Jun Kinoshita, Takahisa Yamaguchi, Hiroto Saito, Daisuke Fujimori, Toshihide Horiike, Shinichi Harada, Hidehiro Tajima, Itasu Ninomiya, Tetsuo Ohta, Sachio Fushida

**Affiliations:** 1 Division of Cancer Medicine, Department of Gastroenterological Surgery, Graduate School of Medical Science, Kanazawa University, 13-1 Takara-machi, Kanazawa, Ishikawa 920-8641 Japan; 2 Center for Biomedical Research and Education, School of Medicine, Kanazawa University, Kanazawa, Ishikawa 920-8641 Japan

**Keywords:** Gastric cancer, Peritoneal dissemination, IL-17A, Mast cell, Fibrosis

## Abstract

**Objectives:**

Interleukin-17A (IL-17A) is pro-inflammatory cytokine and acts as profibrotic factor in the fibrosis of various organs. Fibrosis tumor-like peritoneal dissemination of gastric cancer interferes with drug delivery and immune cell infiltration because of its high internal pressure. In this study, we examined the relationship between IL-17A and tissue fibrosis in peritoneal dissemination and elucidated the mechanism of fibrosis induced by IL-17A using human peritoneal mesothelial cells (HPMCs) and a mouse xenograft model.

**Methods:**

Seventy gastric cancer patients with peritoneal dissemination were evaluated. The correlation between IL-17A and fibrosis was examined by immunofluorescence and immunohistochemistry. A fibrosis tumor model was developed based on subcutaneous transplantation of co-cultured cells (HPMCs and human gastric cancer cell line MKN-45) into the dorsal side of nude mice. Mice were subsequently treated with or without IL-17A. We also examined the effect of IL-17A on HPMCs in vitro.

**Results:**

There was a significant correlation between IL-17A expression, the number of mast cell tryptase (MCT)-positive cells, and the degree of fibrosis (*r* = 0.417, *P* < 0.01). In the mouse model, IL-17A enhanced tumor progression and fibrosis. HPMCs treated with IL-17A revealed changes to a spindle-like morphology, decreased E-cadherin expression, and increased α-SMA expression through STAT3 phosphorylation. Moreover, HPMCs treated with IL-17A showed increased migration.

**Conclusions:**

IL-17A derived from mast cells contributes to tumor fibrosis in peritoneal dissemination of gastric cancer. Inhibiting degranulation of mast cells might be a promising treatment strategy to control organ fibrosis.

## Introduction

Gastric cancer is one of the most common causes of cancer mortality worldwide. A critical factor of poor prognosis and the most common metastatic pattern in gastric cancer is peritoneal dissemination [1–3]. Peritoneal dissemination is characterized by diffusely infiltrating and proliferating cancer cells accompanied by extensive stromal fibrosis in the peritoneal space. The fibrous tissue of peritoneal dissemination causes fatal conditions such as bowel obstruction, jaundice, and hydronephrosis, resulting in aspiration pneumonia, hepatic failure, and renal failure, which are direct causes of death. The prognosis of gastric cancer with peritoneal dissemination is still poor regardless of the various current treatments including systemic chemotherapy [4] and intraperitoneal chemotherapy [5, 6]. Tumor fibrosis interferes with drug delivery and immune cell infiltration because of its high internal pressure [7]. Therefore, control of fibrosis in peritoneal dissemination is necessary to improve treatment outcomes.

Interleukin-17A (IL-17A) is prevalent in various tumor tissues and suppresses tumor immune surveillance. Although it has long been considered that the major cellular source of IL-17A is CD4-positive T lymphocytes (Th17 cells), several studies recently reported that a variety of adaptive and innate immune cell types including γδT cells, natural killer (NK) T cells, NK cells, mast cells, and granulocytes also produce IL-17A [8]. A major source of IL-17A in the primary lesions of gastric cancer is mast cells, and the amount of IL-17A secreted by mast cells is correlated with poor overall survival [9]. In pancreatic ductal adenocarcinoma, esophageal squamous carcinoma, and hepatocellular carcinoma, IL-17A-producing mast cells infiltrate the tumor stroma [10–12]. The role of IL-17A in cancer is thought to correlate with tumorigenesis, tumor proliferation, and angiogenesis [13]. In Crohn’s disease, IL-17A is involved in the development of intestinal fibrosis through epithelial mesenchymal transition (EMT) [14]. A previous study reported a correlation between IL-17A and the degree of tissue fibrosis in systemic sclerosis and lung disease [15]; however, no study has reported a relationship between tissue fibrosis and IL-17A in cancer.

Transforming growth factor (TGF)-β is a common initiator of EMT via the SMAD pathway and it contributes to tissue fibrosis in various organs including the peritoneum [16]. Intraperitoneal free cancer cells from primary gastric lesion first contact and interact with human peritoneal mesothelial cells (HPMCs). We previously reported that TGF-β-mediated activation of HPMCs induces an EMT-like process, and HPMCs activated by TGF-β drive the process of fibrosis by acting as cancer-associated fibroblasts (CAFs) [17]. HPMCs, which are classified as epithelium in the broad sense of the term, form a monolayer of squamous epithelial cells that cover the peritoneal space. Furthermore, HPMCs express the receptor for IL-17A and release chemokines in combination with IL-17A [18]. Some studies reported that the IL-17A pathway in lung adenocarcinoma and gastric cancer cells induces EMT via STAT3 [19–21].

Mast cells contain many cytokines such as TGF-β and IL-17A in their granules and may have various effects on the tumor microenvironment by releasing these cytokines. Additionally, it was reported that mast cells infiltrate the tumor microenvironment via the SCF/c-kit signaling pathway, leading to the aggravation of inflammation and immunosuppression [22].

In this study, we examined which cells are the major source of IL-17A and the relationship between tissue fibrosis and IL-17A in peritoneal dissemination of gastric cancer. Furthermore, we elucidated the mechanism of fibrosis by IL-17A using HPMCs.

## Materials and methods

### Ethics statement

Prior to this research, written informed consent was obtained from each patient. This study was approved by the Institutional Review Board of Kanazawa University Graduate School of Medical Science (No 2789).

### Patients and resource of samples

This study included 70 gastric cancer patients with peritoneal dissemination treated in our department between January 2000 and March 2018. Thirty-six patients underwent combined resection of primary lesions and peritoneal dissemination, whereas 34 patients underwent resection for peritoneal dissemination alone. These 34 patients were confirmed the depth of primary lesions at the time of prior gastrectomy. All samples were obtained before adjuvant therapy. Group A included 29 patients who had obstructive symptoms due to peritoneal dissemination. Group B included 41 patients without bowel obstruction.

### Immunohistochemistry and immunofluorescence

Immunohistochemistry and immunofluorescence were performed as described in a previous study [23]. All sections were stained with hematoxylin and eosin (H&E) and Azan stain to assess fibrosis. For immunohistochemistry, the following primary antibodies were used: mast cell tryptase antibody (ab134932, rabbit monoclonal IgG, diluted 1:100; Abcam, Tokyo, Japan) and IL-17A antibody (AF-317-NA, goat polyclonal IgG, diluted 1:200; R&D Systems, Minneapolis, USA). All sections were examined using a fluorescence microscope (Olympus, Tokyo, Japan). The degree of fibrosis was calculated as the percentage of fibrosis within the whole section using ImageJ software [24]. For immunofluorescence, the following primary antibodies were used: IL-17A antibody (AF-317-NA, goat polyclonal IgG, diluted 1:200; R&D Systems), mast cell tryptase antibody (ab2378, mouse monoclonal IgG, diluted 1:100; Abcam), and CD4 antibody (ab133616, rabbit monoclonal IgG, diluted 1:200; Abcam). The secondary antibodies used were anti-goat IgG antibody conjugated with Alexa Fluor^®^ 594 (ab150132, donkey polyclonal IgG, diluted 1:1000; Abcam), anti-rabbit IgG antibody conjugated with Alexa Fluor^®^ 488 (ab150073, donkey polyclonal IgG, diluted 1:1000; Abcam) and anti-mouse IgG antibody conjugated with Alexa Fluor^®^ 488 (ab150105, donkey polyclonal IgG, diluted 1:1000; Abcam). All slides were then incubated with Hoechst 33258 for nuclear staining. The slides were observed using an immunofluorescence microscope (BX50/BS-FLA; Olympus, Japan). We could confirm double positive (IL-17A and CD4) cells in human tonsil tissue, which was positive control of these antibodies.

### Quantifying immunostaining parameters

Data were obtained by manually counting positively stained cells in three representative regions under 100× high-power magnification. The proportions of double-positive cells per IL-17A-positive cell were calculated. All immunostaining was interpreted by two physicians (SF and JK), who were blinded to the patients’ characteristics.

### Cell lines and cell culture

We isolated HPMCs from surgical specimens of human omentum, as previously described [25]. Donors did not receive chemotherapy or radiation treatment before surgery and had no evidence of peritoneal inflammation and malignancy. The gastric cancer cell line used in this study was MKN-45, which was purchased from the American Type Culture Collection (Rockville, MD, USA). In peritoneal dissemination model, we used the high-potential peritoneal dissemination cell line MKN-45P, which was established from MKN-45 in our institution as described previously [26]. Cells were maintained in RPMI-1640 medium supplemented with 10% fetal bovine serum (FBS).

### Chemicals

Recombinant human IL-17A (AF-200-17) and recombinant mouse IL-17A (210-17) were obtained from Pepro Tech (USA) and reconstituted in RPMI-1640 medium at appropriate concentrations. Sttatic STAT3 inhibitor (ab120952) was purchased from Abcam and reconstituted in DMSO to 50 mM.

### Mouse xenograft model

All animal experiments were performed according to Kanazawa University’s standard guidelines. Female immunocompromised BALB/c-nu/nu mice (Charles River Laboratories Inc., Tokyo, Japan) at 4–6 weeks of age were maintained in a sterile environment. In the subcutaneous tumor model, MKN-45 cells were co-cultured with an equivalent number of HPMCs for 5 days, and a total of 5 × 10^6^ cells in 100 µl RPMI-1640 were subcutaneously implanted into the dorsal side of each mouse on day 0. We established two groups of ten mice each with or without IL-17A. In the IL-17A-treated group, recombinant human IL-17A (AF-200-17) was intraperitoneally administered daily at 3 µg/mouse from day 5 to day 10. Animals were carefully monitored, and tumors were measured every 2 days. At day 14, the mice were killed, and tumors were removed for immunohistochemical examination. In peritoneal dissemination model, a total of 5 × 10^6^ MKN-45P cells were intraperitoneally injected into nude mice on day 0. The scratch method of i.p. cell inoculation was employed [27]. We established two groups of three mice each. In the IL-17A-treated group, recombinant mouse IL-17A (210–17) was intraperitoneally administered daily at 3 µg/mouse from day 1 to day 5. At day 14, the mice were killed, and tumors were removed. The tumor volume (*V*) was calculated according to the formula *V* = *AB*^2^/2, where *A* is the length of the major axis and *B* is the length of minor axis. Tumor specimens were stained with H&E and Azan staining. The expression of α-SMA antibody (ab5694, rabbit polyclonal IgG; diluted 1:200; Abcam) was assessed immunohistochemically. The degree of fibrosis and α-SMA-positive areas were calculated as the percentage of fibrosis and the α-SMA-positive area within the whole section using ImageJ software [24].

### Phase contrast microscopy

HPMCs were seeded into 100-mm tissue culture dishes at 5.0 × 10^4^ cells in RPMI-1640 growth medium with 10% FBS. HPMCs in culture were treated with IL-17A (100 ng/ml) for 72 h and morphological changes were visualized by phase contrast microscopy. The images were captured using an inverted microscope (Nikon Corp., Japan).

### Immunocytochemistry

Cells were grown on four-well collagen type I-coated culture slides (BD BioCoat). The slides were incubated with fibroblast activation protein (FAP) antibody (ab53066, rabbit polyclonal IgG: diluted 1:100; Abcam), α-SMA antibody (1A4, mouse monoclonal IgG; diluted 1:100; DakoCytomation, Denmark), and E-cadherin antibody (H-108, rabbit polyclonal IgG; diluted 1:100; Santa Cruz Biotechnology, Inc., USA). The secondary antibodies were anti-rabbit IgG antibody conjugated with Alexa Fluor^®^ 488 (ab150073, donkey polyclonal IgG, diluted 1:1000; Abcam) and an anti-mouse IgG antibody conjugated with Alexa Fluor 594^®^ (ab150116, goat polyclonal IgG, diluted 1:1000; Abcam). The slides were observed using an immunofluorescence microscope (BX50/BS-FLA; Olympus, Japan).

### Western blotting

Protein from each sample was loaded onto 12.5% SDS-PAGE gels and subjected to electrophoresis. Proteins were transferred to PVDF membranes (Bio-Rad, USA) and blocked with blocking solution (0.1% Tween-20; EZ Block ATTO Corporation, Japan). Blots were incubated overnight at 4 ℃ with each primary antibody. The blots were incubated with appropriate HRP-conjugated secondary antibodies and visualized using an ECL Plus western blotting detection system (GE Healthcare Japan Ltd., Japan) and the Light-capture system (ATTO). To ensure equal protein loading, β-actin levels were measured using an anti-β-actin monoclonal antibody (AC-15, mouse monoclonal IgG, diluted 1:10,000; Sigma). The following primary antibodies were used: E-cadherin (4A2C7, mouse monoclonal IgG, diluted 1:1000; Thermo Fisher Scientific, USA), Vimentin (V9, mouse monoclonal IgG, diluted 1:1000; Santa Cruz Biotechnology, Inc., USA), α-SMA (1A4, mouse monoclonal IgG; diluted 1:2000; DakoCytomation), ZEB-1 (ab203829, rabbit monoclonal IgG, diluted 1:500; Abcam), STAT3 (D3Z2G, rabbit monoclonal Biotinylated, diluted 1:1000; Cell Signaling Technology, USA), phospho-STAT3 (D3A7, rabbit monoclonal IgG, diluted 1:2000; Cell Signaling Technology), SMAD2/3 (C-8, mouse monoclonal IgG, 1:1000; Santa Cruz Biotechnology, Inc.), and phospho-SMAD2/3 (Ser423/425, goat polyclonal IgG, diluted 1:1000; Santa Cruz Biotechnology, Inc.).

### MTT assay

HPMCs were seeded in 96-well plates at a density of 4 × 10^3^ cells per well in RPMI-1640 growth medium with 10% FBS. After incubation, supernatant was discarded and replaced with fresh serum-free medium, and various concentrations of IL-17A (50, 100, 200, 500 ng/ml) were added. At 48 h post-treatment, supernatant was discarded and 3-(4,5-dimethylthiazol-2-yl)-2,5-diphenyltetrazolium bromide (MTT) solution was added to all wells (final concentration, 500 µg/ml). The supernatant was removed and 150 µL DMSO (Wako, Japan) was added. The absorbance of the solution in each well was measured at 535 nm using a microplate reader (model 550; Bio-Red, Japan). The cell viability was calculated as viability = (absorbance of experimental wells)/(absorbance of control wells). All experiments were repeated three times.

### Wound healing assay

HPMCs were cultured as confluent monolayers in a six-well plate and were wounded by removing a 1-mm strip across the well with a pipette tip. The wounded monolayer was washed with PBS to remove non-adherent cells before 1 ml IL-17A (100 ng/ml) (AF-200-17, Pepro Tech, USA) or vehicle control was added. After 0, 6, 12, and 24 h incubation, the distance of the wound was imaged by inverted microscopy (Nikon Corp., Japan) and measured.

### Invasion assay

For invasion assays, we used a BD BioCoat Matrigel Invasion Chamber for 24-well plates (BD Bioscience, USA), according to the manufacturer’s instructions. First, Matrigel was rehydrated using 750 μl serum-free medium, after which we added 750 μl of fresh medium containing 10% FBS to the lower chamber with or without IL-17A (100 ng/ml). Next, 0.5 ml HPMCs (1 × 10^5^ cells/ml) in serum-free media were seeded into the upper chamber of the system. After 48 h of incubation, the cells in the upper chamber were removed and the cells that had invaded through the Matrigel membrane were stained with hematoxylin. Membranes were removed from inserts and mounted on slides. Invading cells were counted by microscopy under 100× high-power magnification in several fields of triplicate membranes.

### Statistical analysis

All data are expressed as the mean ± SE. Statistical analyses were conducted using SPSS statistical software, version 23 (IBM Corp., Armonk, NY, USA). All data of patients’ characteristics were analyzed by Chi-square test. Comparisons of the numbers of IL-17A-positive cells, MCT-positive cells, and double-positive cells were made using the Student’s *t* test. The degree of fibrosis was compared using the Student’s *t* test. The correlation between fibrosis and the number of mast cells producing IL-17A was assessed using Spearman’s correlation coefficient. Comparisons of drug effects were made using Mann–Whitney *U* test. A *p* value of < 0.05 indicated statistical significance.

## Results

### Patients’ characteristics

Patients’ characteristics are shown in Table 1. Overall, 34 men and 36 women were enrolled. The age of the study population ranged from 40 to 83 (median, 58.9) years. Twenty-nine patients had obstructive symptoms related to peritoneal dissemination. Group A consisted of 29 patients with bowel obstruction and group B consisted of 41 patients who had no symptoms related to peritoneal dissemination. There were no significant differences between group A and group B for patients’ characteristics. In both group A and B, the primary histological features were mainly diffuse type, small stroma and penetration depth was tumor penetration of serosa (SE) or tumor invasion of adjacent structures (SI). These 34 patients were confirmed the depth of primary lesions at the time of prior gastrectomy.

**Table 1:** Patients’ characteristics

	Group A *n* (%)	Group B *n* (%)	*p* value
Gender			0.967
Male	14 (48.3)	20 (48.9)	
Female	15 (51.7)	21 (51.1)	
Age			0.290
≦60	14 (48.3)	14 (34.1)	
>60	15 (51.7)	27 (65.9)	
Histological type of primary lesion			0.810
Intestinal	5 (17.2)	8 (19.5)	
Diffuse	24 (82.8)	33 (80.5)	
Scirrhous stroma of primary lesion			0.100
Yes	6 (20.7)	3 ( 7.3)	
No	23 (79.3)	38 (92.7)	
Depth of primary lesion			0.796
SS	7 (24.1)	10 (24.4)	
SE or SI	22 (75.9)	31 (75.6)	
Gastrectomy			0.352
Yes	13 (44.8)	23 (56.0)	
No	16 (55.2)	18 (44.0)	
Adjuvant therapy before taking samples			–
Yes	0 ( 0)	0 ( 0)	
No	29 (100)	41 (100)	

*Group A* obstructive symptoms (+), *Group B* obstructive symptoms (−), *SE* tumor penetration of serosa, *SI* tumor invasion of adjacent structure, *SS* subserosa

### Mast cells are the main producers of IL-17A in peritoneal dissemination

**Fig. 1:**
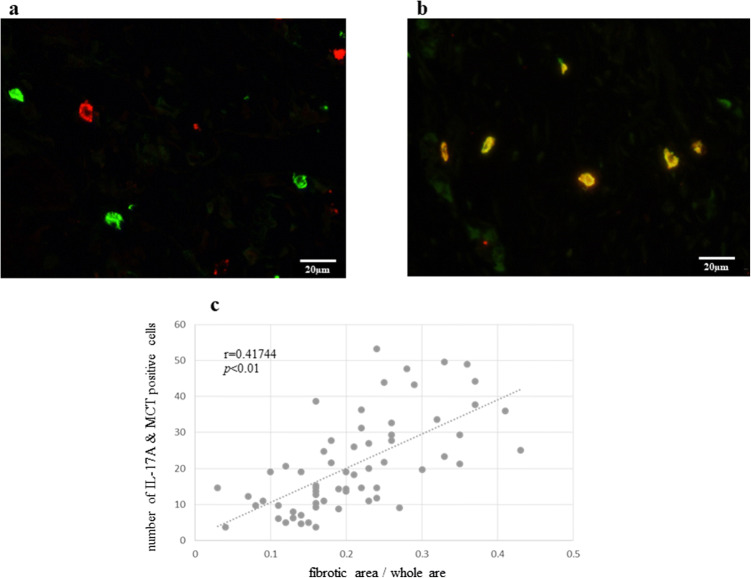
**a** IL-17A positive cells are red and CD4-positive cells are green. There were no double-positive cells (IL-17A and CD4) in the peritoneal disseminated lesion (original magnification ×400). **b** Double positive (IL-17A and MCT) cells are orange. Mast cells highly expressed IL-17A in the peritoneal dissemination (original magnification ×400). **c** The number of double (IL-17A and MCT)-positive cells correlated with the degree of fibrosis in peritoneal dissemination. *p* values were determined by Spearman’s correlation coefficient

Many IL-17A-positive cells and MCT-positive cells were present in peritoneal dissemination. To identify the IL-17A-producing cells in peritoneal dissemination, we performed double immunofluorescence staining using IL-17A and CD4 antibodies. Although CD4 is one of the surface markers for Th17 cells, which produce IL-17A in various tissues, there were no IL-17A/CD4 double-positive cells in peritoneal dissemination tissues (Fig. 1a). However, double immunofluorescence staining for IL-17A and MCT indicated many double-positive cells (Fig. 1b). Approximately 70% of IL-17A-producing cells were MCT-positive.

### IL-17A secreted by mast cells is associated with the degree of fibrosis in peritoneal dissemination

Patients were divided into two groups based on the presence or absence of obstructive symptoms. The mean number of immune reactive cells against IL-17A and MCT in group A was significantly greater than that in group B (Table 2). The degree of tissue fibrosis between the two groups was compared by Azan staining. A comparison of the fibrotic area showed that group A with obstructive symptoms had significantly more fibrotic areas than group B (Table 3). The number of double (IL-17A and MCT)-positive cells correlated with the degree of fibrosis in peritoneal dissemination (*r* = 0.417, *p* < 0.01; Fig. 1c).

**Table 2:** Number of IL-17A- and MCT-positive cells in the peritoneal dissemination

	Group A	Group B	*p* value
IL-17A positive	5.2 ± 11.5	17.8 ± 6.4	<0.001
MCT positive	45.8 ± 11.4	17.8 ± 7.2	<0.001
IL-17A and MCT positive	34.6 ± 10.8	12.3 ± 6.2	<0.001

Group A included patients with bowel obstruction and group B included patients without bowel obstruction. Date are shown as mean ± SD

*MCT* mast cell tryptase

**Table 3:** Ratio of fibrotic area in the peritoneal dissemination

	Group A	Group B	*p* value
Fibrotic area/whole area (mean ± SD)	0.294 ± 0.07	0.167 ± 0.05	<0.001

### Effect of IL-17A in subcutaneous xenograft models

**Fig. 2:**
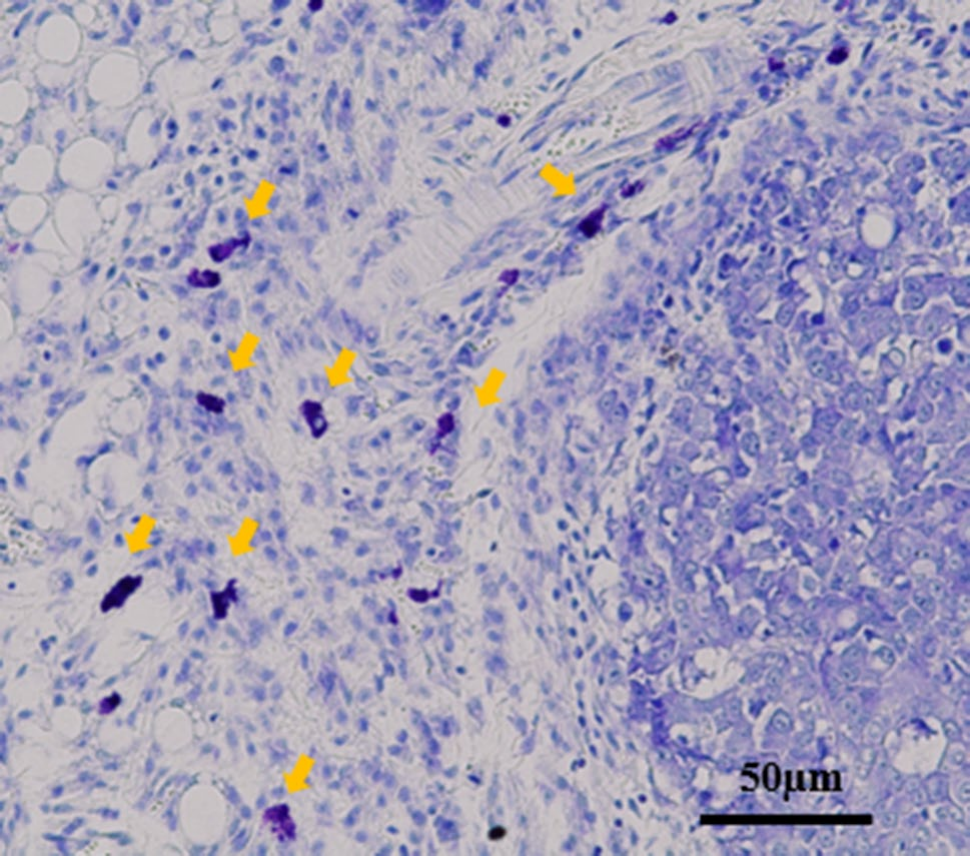
Mast cells derived from host nude mouse were found in subcutaneous tumor. Mast cells were recognized by toluidine blue staining (original magnification ×200)

**Fig. 3:**
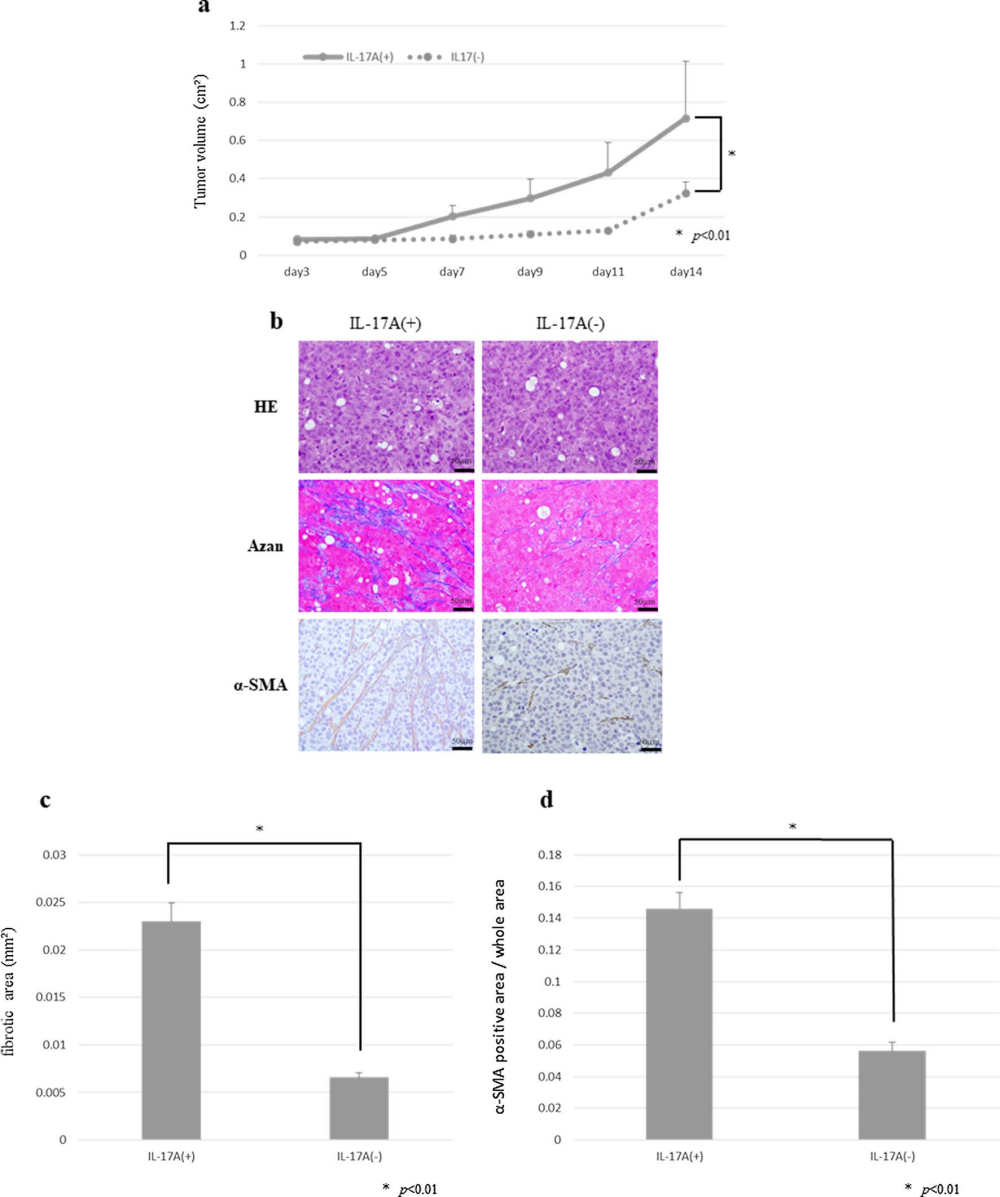
Subcutaneous xenograft model to investigate the effect of IL-17A. **a** Time course of tumor growth at day 14 in the IL-17A-treated group and the control group. Results are expressed as the mean ± SD (*n* = 10). **b** Microscopic view of mouse xenograft tumors. Histological examination using H&E staining. Fibrous tissue was determined by Azan staining. Immunohistochemical examination of α-SMA was performed. **c** Fibrous tissue was measured. Data are expressed as the mean ± SD in three representative regions at ×200 high-power magnification. **d** A α-SMA positive lesion was measured and is shown as a percentage (α-SMA-positive area/whole section area). Data are expressed as the mean ± SD in three representative regions at ×200 high-power magnification

We also confirmed mast cells infiltration into subcutaneous fibrous tumor stained by toluidine blue (Fig. 2). To examine the effect of IL-17A in vivo, a dose of 3 µg/body/day was administered intraperitoneally to nude mice with tumor xenografts. The time-dependent changes in subcutaneous tumor volume are shown in Fig. 3a. At 14 days after transplantation, the mean tumor volume increased significantly in the IL-17A-treated group compared with that in the control group. Histological and immunohistochemical examinations revealed that the tumors derived from the IL-17A-treated group had larger areas of fibrosis (Fig. 3b, c) and enhanced α-SMA expression (Fig. 3b, d).

### Effect of IL-17A in peritoneal dissemination models

**Fig. 4:**
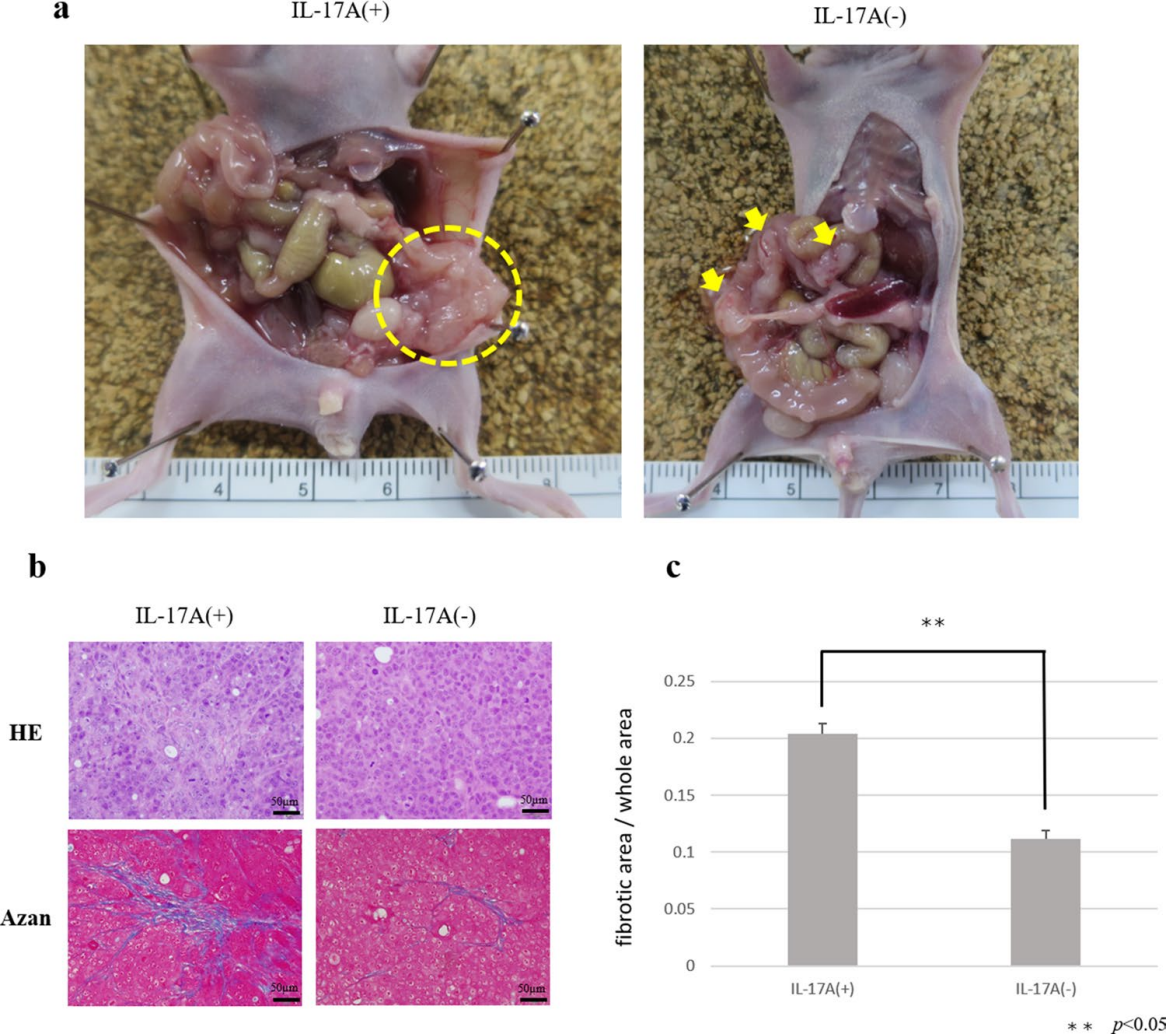
Peritoneal dissemination model to investigate the effect of IL-17A. **a** Macroscopic views of peritoneal nodules (arrow head). **b** Microscopic view of tumors. Histological examination using H&E staining. Fibrous tissue was determined by Azan staining. **c** Fibrous tissue was measured and is shown as a percentage (fibrotic area/whole area). Data are expressed as the mean ± SD in three representative regions at ×200 high-power magnification

Control group without IL-17A showed small nodules scattered in mesentery. On the other hand, MKN-45P with IL-17A inoculated group showed large tumor invaded into intestinal tract (Fig. 4a). The ratio of fibrotic areas in IL-17A-treated tumors was significant higher than in non-treated tumors (Fig. 4b, c).

### HPMCs showed morphological changes and expression of EMT markers by IL-17A

**Fig. 5:**
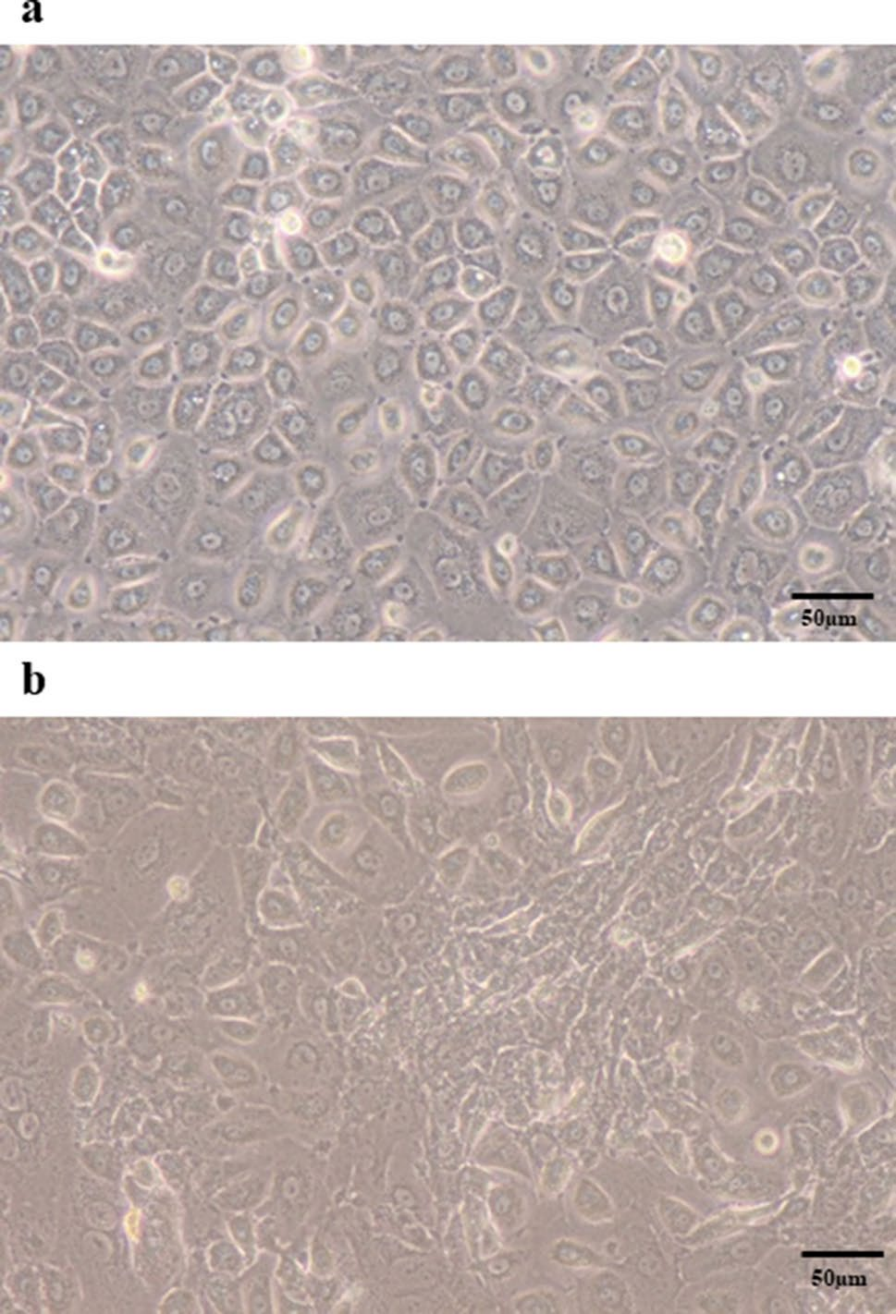
Phase contrast microscopy of morphological changes in HPMCs (original magnification ×400). **a** HPMCs cultured in control medium showed a cobblestone-like growth pattern. **b** HPMCs treated with IL-17A (100 µg/ml) for 48 h exhibited a spindle-shape morphology characteristic of fibroblasts

**Fig. 6:**
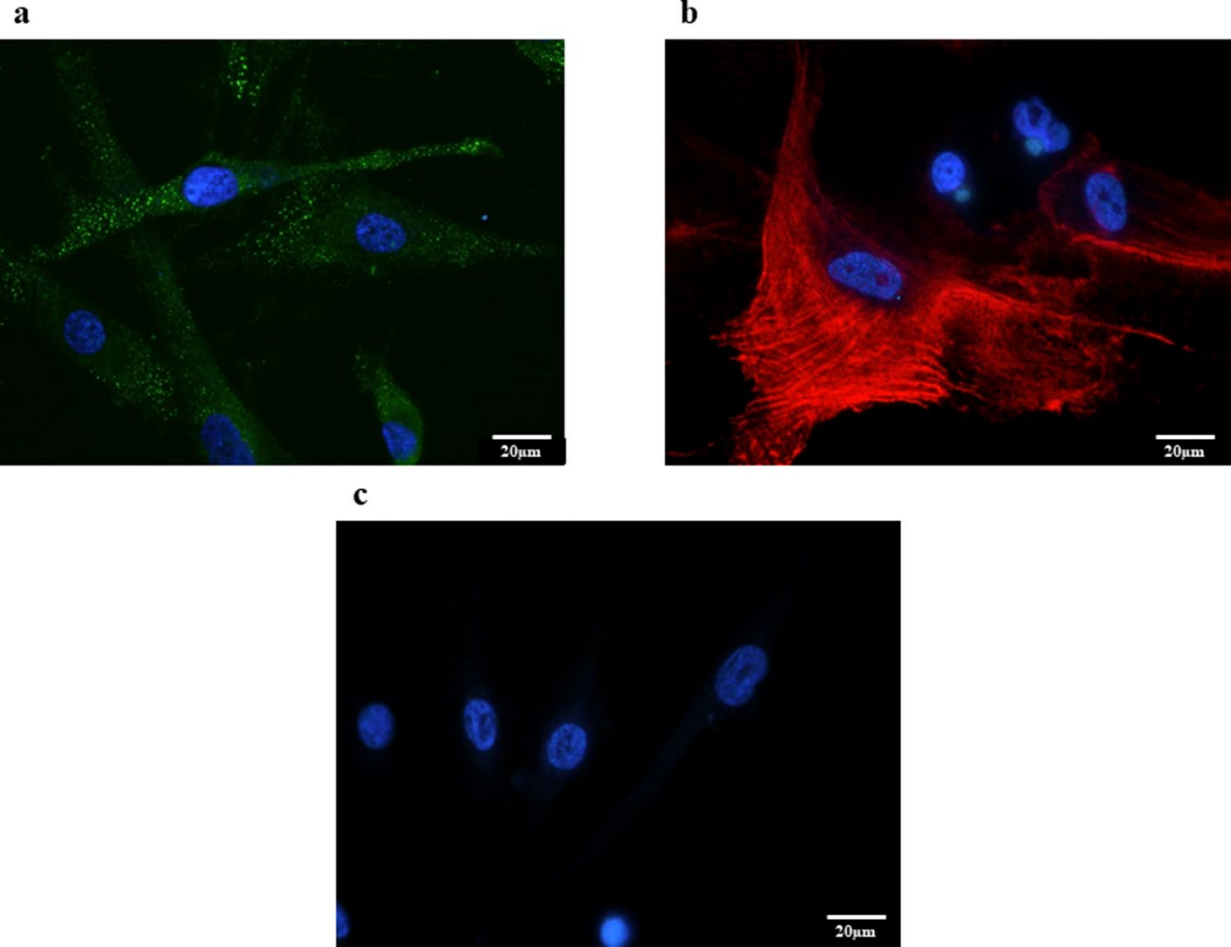
**a** Representative photomicrographs of immunofluorescence staining for FAP. Expression of FAP was observed in HPMCs treated with IL-17A (original magnification ×400). **b** Representative photomicrographs of immunofluorescence staining for α-SMA. Expression of α-SMA was observed in HPMCs treated with IL-17A (original magnification ×400). **c** Representative photomicrographs of immunofluorescence staining for E-cadherin. Expression of E-cadherin was not observed in HPMCs treated with IL-17A (original magnification ×400)

**Fig. 7:**
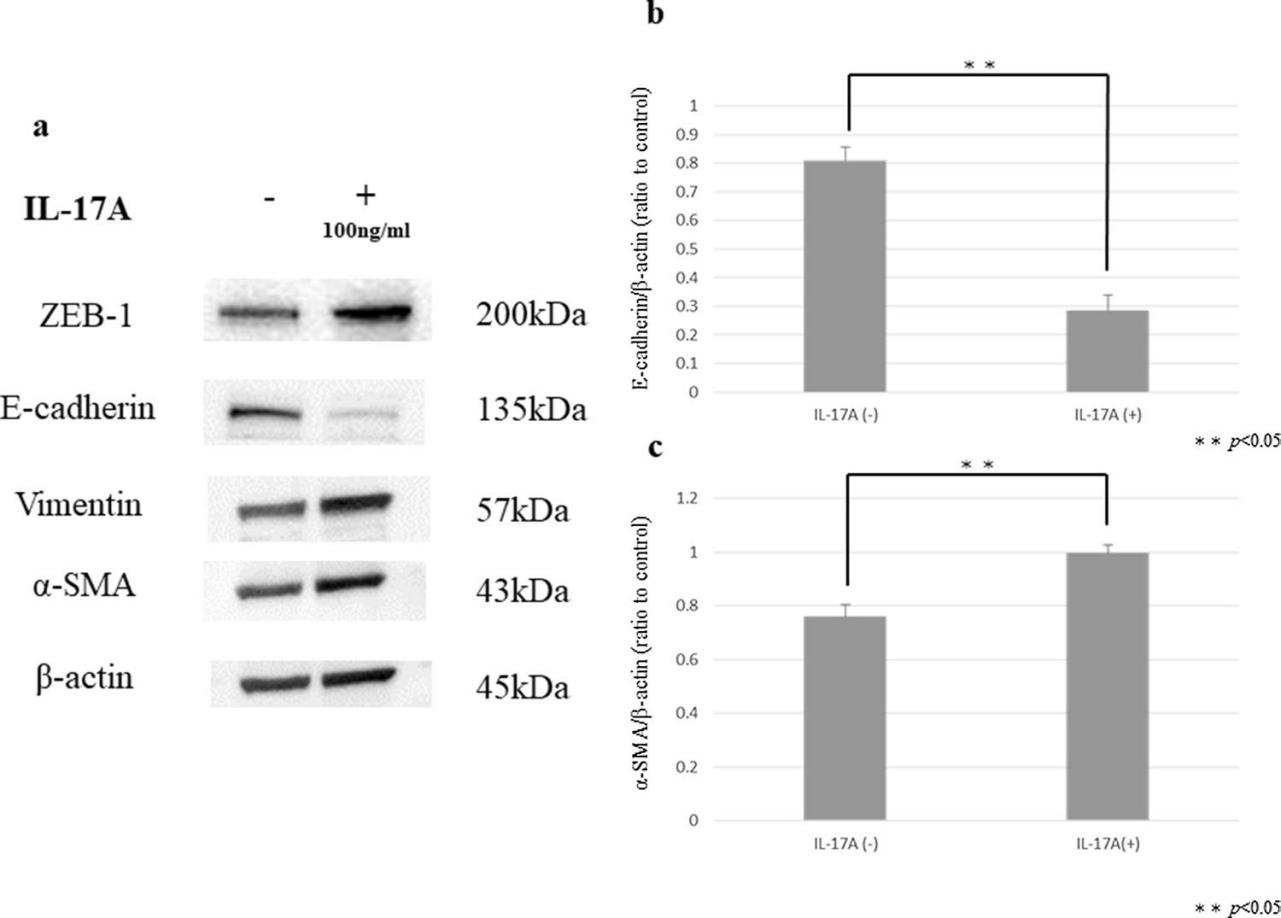
Western blot analysis of ZEB-1, E-cadherin, Vimentin, and α-SMA expression in HPMCs. **a** ZEB-1, Vimentin, and α-SMA expression were higher in HPMCs treated with IL-17A than in the control. E-cadherin expression was attenuated in HPMCs treated with IL-17A. **b**, **c** Densitometry analyses of E-cadherin and α-SMA were performed in three independent experiments; data are expressed as the mean ± SD

Untreated HPMCs showed a polygonal and cobblestone-like growth pattern (Fig. 5a). However, HPMCs treated with IL-17A (100 ng/ml) exhibited the spindle-shaped morphology that is characteristic of fibroblasts (Fig. 5b). FAP expression was observed by immunofluorescence staining of HPMCs treated with IL-17A (Fig. 6a). The expression of the mesenchymal marker α-SMA was increased and E-cadherin expression was decreased after administration of IL-17A (Fig. 6b, c). Western blotting also confirmed the increased α-SMA expression and decreased E-cadherin expression in HPMCs treated with IL-17A. Additionally, western blotting showed that the mesenchymal markers Vimentin and ZEB-1 were upregulated (Fig. 7a–c).

### Effect of IL-17A on the proliferation of HPMCs

**Fig. 8:**
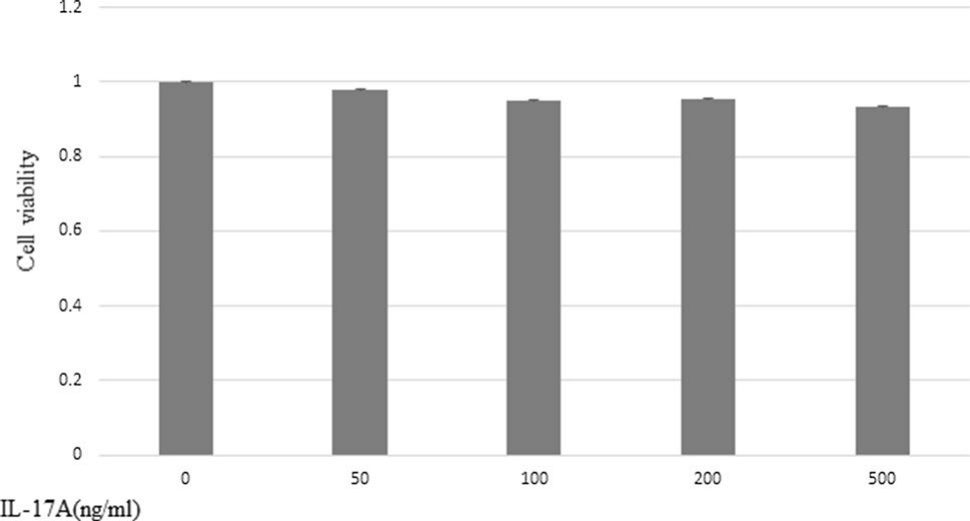
Effect of IL-17A on the proliferation of HPMCs. MTT assays showed no significant difference in HPMC proliferation at each concentration of IL-17A. Data are presented as the mean ± SD of three independent experiments

There was no significant difference in the proliferative capacity of HPMCs treated with IL-17A at each concentration (0, 50, 100, 200, 500 ng/ml) (Fig. 8).

### HPMCs treated with IL-17A had increased ability of migration

**Fig. 9:**
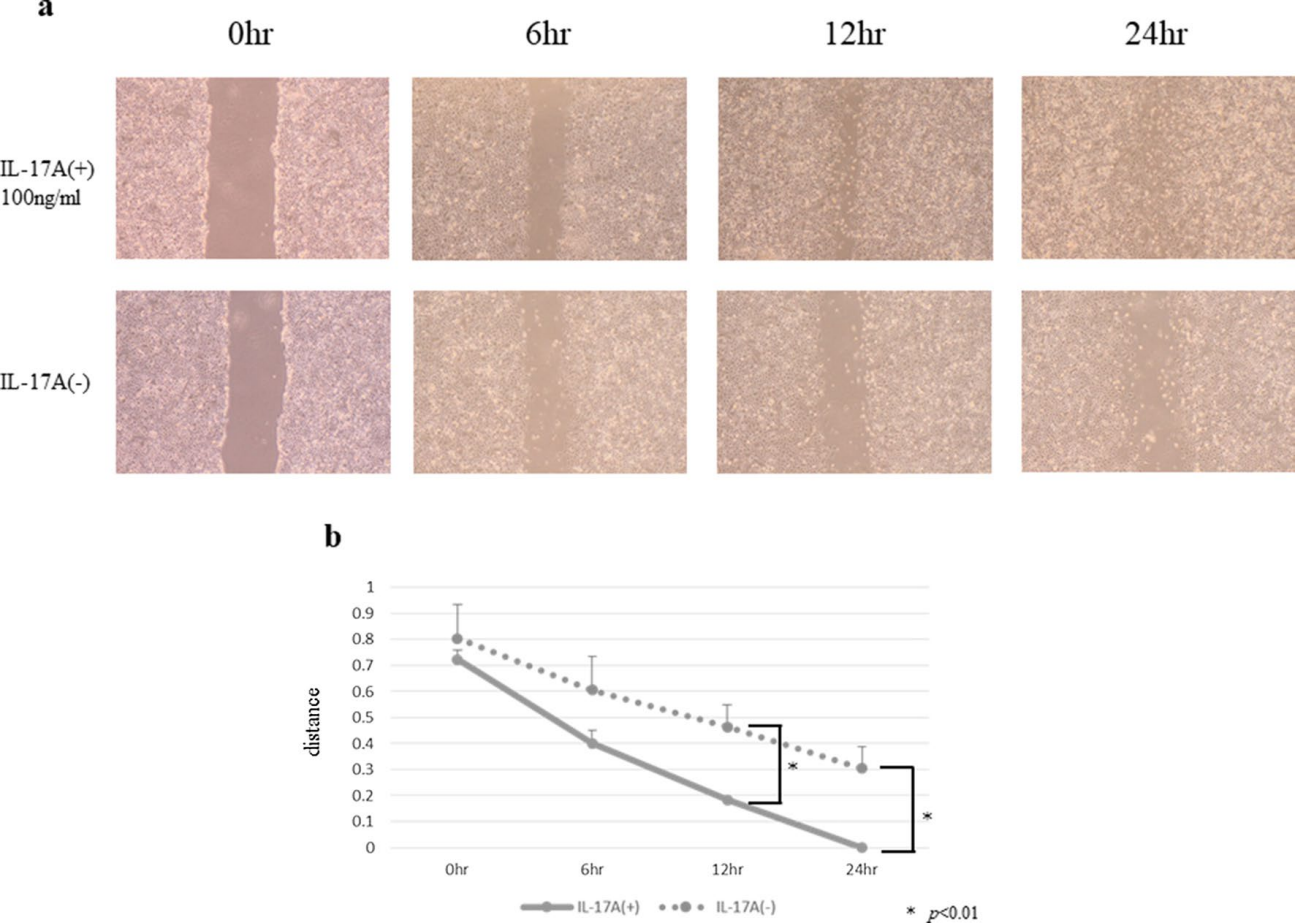
HPMCs were incubated with or without IL-17A for 24 h (IL-17A, 100 ng/ml). **a** Images were acquired by microscopy at several time points (original magnification ×40). **b** IL-17A significantly promoted wound healing at 12 and 24 h. Data are presented as the mean ± SD of three independent experiments

**Fig. 10:**
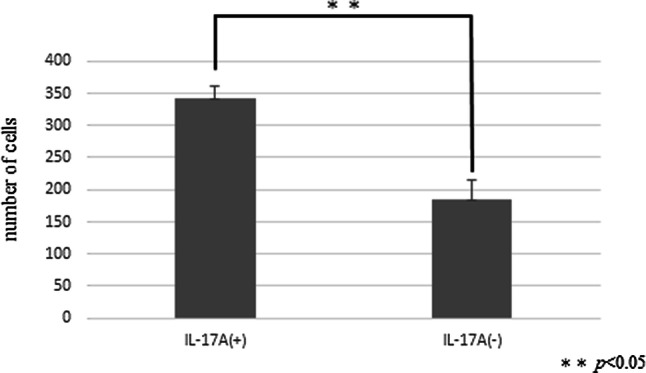
Effect of IL-17A on the invasiveness of HPMCs. IL-17A significantly promoted the invasion ability of HPMCs. Data are presented as the mean ± SD of three independent experiments

The wound healing assay showed that IL-17A markedly and persistently promoted HPMC wound closure at several time points (Fig. 9a, b). In the invasion assay, the ability to migration was upregulated in HPMCs treated with IL-17A (Fig. 10).

### IL-17A promoted the EMT-related transformation of HPMCs through the STAT3 pathway

**Fig. 11:**
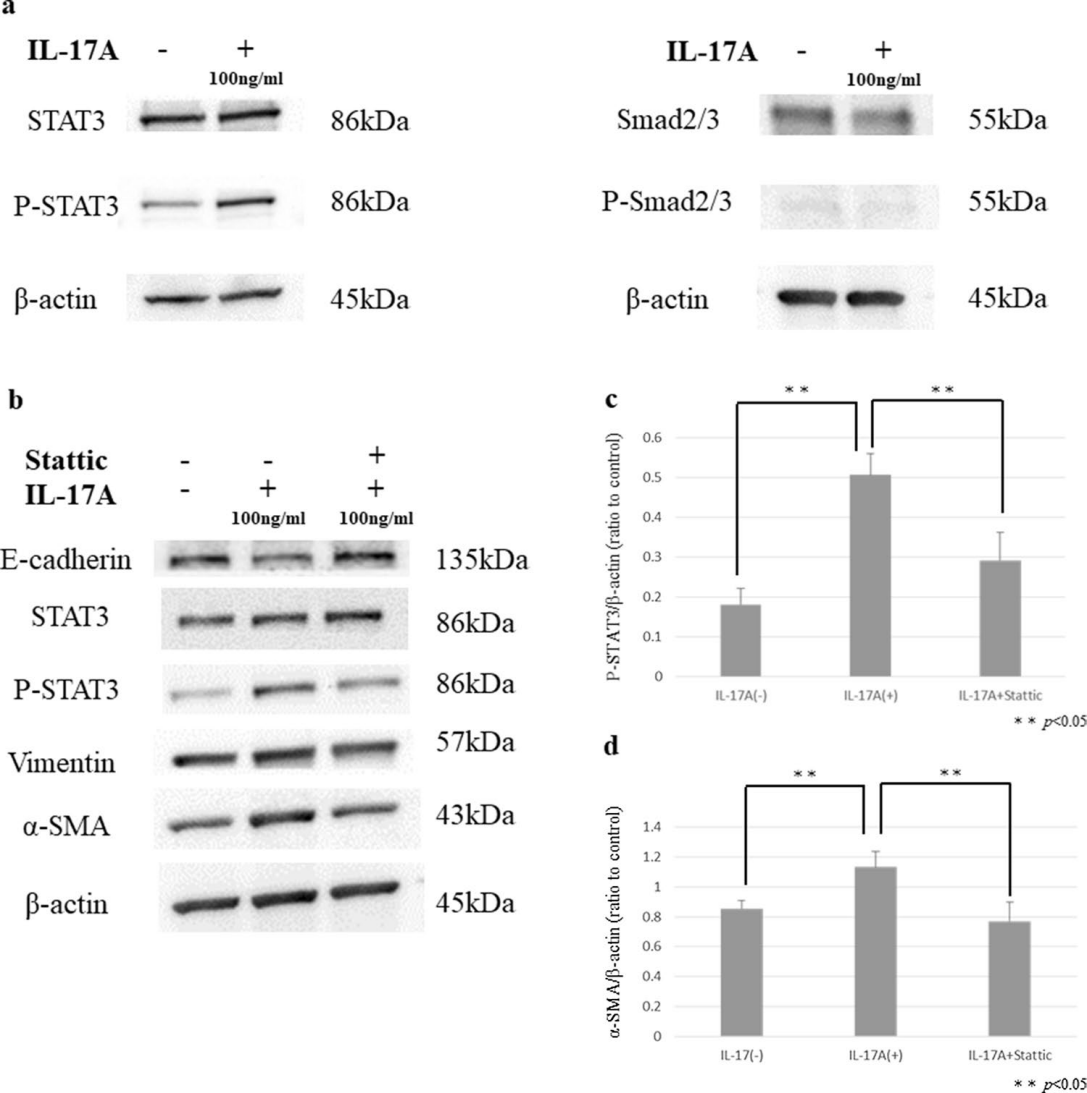
**a** P-STAT3 expression was higher in HPMCs treated with IL-17A than in the control. There was no increase in the expression of p-Smad2/3 in either the IL-17A-treated group or the control group. **b** E-cadherin expression was higher in HPMCs treated with IL-17A and Sttatic than those treated with IL-17A only. P-STAT3, Vimentin, and α-SMA expression was attenuated in HPMCs treated with IL-17A and Sttatic. **c**, **d** Densitometry analyses of p-STAT3 and α-SMA were performed in three independent experiments; data are expressed as the mean ± SD

Western blot analysis showed that IL-17A increased the expression of phospho-STAT3 in HPMCs. However, IL-17A had no effect on the expression of phospho-SMAD2/3 in HPMCs (Fig. 11a). Sttatic is a selective STAT3 inhibitor that inhibits the activation, dimerization, and nuclear translocation of STAT3 by interacting with its SH2 domain. Co-administration of Sttatic inhibited the enhanced expression of EMT markers such as a-SMA and Vimentin by IL-17A in HPMCs (Fig. 11b–d).

## Discussion

In the present study, we showed that mast cells are the major source of IL-17A in peritoneal dissemination of gastric cancer. Moreover, the number of IL-17A-producing cells correlated with tissue fibrosis. In a mouse fibrosis model, intraperitoneal administration of IL-17A promoted tumor growth and intratumoral fibrosis. In this in vitro study, IL-17A induced EMT-like changes in HPMCs. Thus, IL-17A attenuated E-cadherin expression and increased α-SMA expression in HPMCs via the STAT3 pathway, and these changes were suppressed by Sttatic, an inhibitor of STAT3. These results suggest that IL-17A derived from mast cells induced HPMCs to CAFs in peritoneal dissemination, resulting in abundant stromal fibrosis.

EMT is considered as the critical step for tissue fibrosis by which epithelial cells gain greater invasive and migration abilities. Several experiments reported that EMT can be induced not only by loss of cellular contact (for example, due to degradation of basement membranes), but also by various cytokines, especially by TGF-β [28]. We have previously reported that TGF-β-mediated activation of HPMCs and transformation into CAFs promoted peritoneal fibrosis [17]. In this study, we clarified that IL-17A also has an important role in tissue fibrosis as well as TGF-β. IL-17A induced EMT-like changes in HPMCs via STAT3, and not the TGF-β/SMAD pathway. IL-17A is positively correlated with the activation of the STAT3 signaling pathway [29]. The activation of STAT3 through the phosphorylation of Tyr705 is facilitated by the JAK signaling pathway [30, 31]. In this in vitro study, STAT3 inhibition by Sttatic suppressed the EMT-like changes in HPMCs induced by IL-17A. Although inhibiting the IL-17A/STAT3 pathway may allow control of tissue fibrosis, molecular targeting drugs for IL-17A, such as secukinumab and iexkizumab, are expensive and have not been used for malignant tumors. Therefore, we focused on mast cells, which are the main producers of IL-17A in peritoneal dissemination. Mast cells have been shown to accumulate in adenomatous polyps (precursors to invasive colon cancer) [32] and many developing tumors, particularly malignant melanoma [33], breast cancer [34], and colorectal carcinoma [35]. According to previous studies of mast cell accumulation in tumors, mast cells present mainly at the tumor periphery, or at the border with normal tissue, rather than within the tumor [36, 37]. Peripheral mast cell localization indicates that recruitment occurs either from (a) resident mast cells migrating from neighboring healthy tissue or, (b) de novo recruitment of mast cells progenitors via normal vasculature close to the tumor site (but not through tumor vasculature), or both [38]. Recently, stem cell factors (SCFs) produced by tumor cells were implicated in mast cell accumulation in the periphery of tumors [22, 39]. During the development of peritoneal dissemination, cancer cells released from the primary lesion adhere to the peritoneum covered with HPMCs. Mast cells accumulate around tumor cells due to SCFs and degranulate and release cytokines such as IL-17A, affecting tumor progression and fibrosis. Dense fibrosis confers elevated intratumoral pressure and solid stress, resulting in vascular compression and reduced diffusion into the tumor interstitium [7]. To control the fibrosis, it may be necessary to reduce IL-17A secretion by suppressing degranulation of mast cells, which are the main producers of IL-17A in peritoneal dissemination. Mast cells contain not only IL-17A but also TGF-β in their granules [40]. We have previously demonstrated that tranilast, which is one of anti-allergic drugs, acted HPMCs resulted in inhibition of tumor growth and fibrosis by suppressing Smad2/3 phosphorylation of the TGF-β/Smad pathway [41]. Tranilast also inhibits degranulation containing IL-17A from mast cells. Accordingly, antifibrotic function of tranillast in mouse subcutaneous tumor is thought to be due to the above two effects. Generally, IL-17A is secreted from Th17 cells and systemic inhibition of IL-17A may induce immune-related adverse events. Thus, it is considered to be effective to suppress the function of mast cells, and could therefore be expected in controlling fibrosis in peritoneal dissemination.

We demonstrated IL-17A-producing mast cells correlated with tumor fibrosis, and elucidated that IL-17A induced CAFs from mesothelial cells in vitro and is involved in tumor fibrosis in vivo. However, this study had some limitations. In studies using clinical specimens, our study was small, further larger specimens are required to validate our results. CAFs also affects cell-mediated immune resistance in the cancer microenvironment, thus it is necessary to understand the effect of IL-17A using immunocompetent mouse in near future.

In conclusion, the major source of IL-17A is mast cells, and IL-17A contributes to tumor progression and fibrosis in the peritoneal dissemination of gastric cancer. The control of organ fibrosis might decrease intratumoral pressure, resulting in increased drug delivery and immune cell infiltration. Chemo-immune therapy combined with the suppression of mast cell degranulation might be a promising treatment strategy for gastric cancer patients with peritoneal dissemination.
